# Effectiveness of distraction-based interventions for relieving anxiety, fear, and pain in hospitalized children during venous blood sampling: a systematic review protocol

**DOI:** 10.11124/JBIES-22-00057

**Published:** 2023-11-06

**Authors:** Saija Huhtala, Anna-Kaija Palomaa, Anna-Maria Tuomikoski, Tarja Pölkki

**Affiliations:** 1 Kajaani University of Applied Sciences, Kajaani, Finland; 2 Research Unit of Health Sciences and Technology, University of Oulu, Oulu, Finland; 3 Medical Research Center Oulu, University Hospital and University of Oulu, Oulu, Finland; 4 Oulu University Hospital, Oulu, Finland; 5 Finnish Centre for Evidence-Based Health Care: A JBI Centre of Excellence, Nursing Research Foundation, Helsinki, Finland

**Keywords:** anxiety, children, fear, pain management, venipuncture procedure

## Abstract

**Objective:**

The aim of this review is to evaluate the effectiveness of active compared with passive distraction-based interventions for relieving anxiety, fear, and pain in hospitalized preschool and school-age children during venous blood sampling.

**Introduction:**

Venous blood sampling remains the most common procedure that causes anxiety, fear, and pain among the pediatric population. It is important that health care professionals relieve a child’s pain and the related emotions because untreated pain may have long-term effects on children’s growth and development. It is necessary to determine which interventions are effective in relieving these outcomes in preschool and school-age children during blood sampling.

**Inclusion criteria:**

This review will include randomized controlled trials and quasi-experimental studies that include active and passive distraction-based interventions for relieving hospitalized preschool and school-age children’s anxiety, fear, and pain during venous blood sampling.

**Methods:**

CINAHL, PubMed, Scopus, and the Cochrane Library databases will be searched for published studies. MedNar, Google Scholar, and PsycEXTRA databases will be searched for in-progress and unpublished studies. Two independent researchers will perform critical appraisal and data extraction using the JBI methodology. Data describing randomized controlled trials and quasi-experimental studies will be pooled in a statistical meta-analysis. If statistical analysis is not possible, the findings will be reported narratively. The Grading of Recommendations Assessment, Development, and Evaluation (GRADE) approach will be used to assess certainty in the quality of evidence.

**Review registration:**

PROSPERO CRD42023455617

## Introduction

Venous blood sampling remains the most common procedure that causes anxiety, fear, and pain among hospitalized preschool and school-age children.^1–5^ Children with long-term illness, in particular, are exposed to the pain associated with repeated blood sampling due to the treatment and follow-up their illness requires. Blood samples are often taken intravenously from hospitalized children^1–5^ because the venous blood sampling method is somewhat less painful than other procedures (eg, finger prick) and allows for high-quality samples from the child.

Anxiety, fear, and pain in hospitalized children during venous blood sampling is related to the child’s age, and younger children are often more afraid than older children. Studies have shown that age is inversely related to reported needle pain and fear, which decreases with age.^6^ Of children under the age of 9, 75% are afraid of needlesticks,^6^ while 33% of 9-year-old children almost always fear blood sampling.^7^ Anxiety and fear are associated with a child’s pain during venous blood sampling because these feelings can cause physiological, psychological, and emotional effects in children.^1,2,4,5,8^ Needle-related procedures can cause anxiety and fear, which can manifest as an increased heart rate and fear of nurses.^9^ Children may cry, become angry, or exhibit negative behaviors toward their parents for weeks after a frightening and painful procedure.^10^ Moreover, bad experiences related to untreated pain can have long-term negative effects on children’s growth and development (eg, their emotional state).^11^

In the context of children’s sense of safety, anxiety and fear are closely related concepts. Anxiety describes concern about future events, whereas fear is directed at an immediately perceptible stimulus.^12^ Separation from parents causes anxiety among hospitalized preschool and school-age children, whereas procedures, along with the pain associated with them, are a source of fear.^12^ Factors that predispose children to anxiety and fear in hospital include age, hospital experiences, social incompatibility, inadequate coping, and parental anxiety.^8^ Children’s pain is a complex phenomenon because pain is a subjective experience.^8^ Providing a universal definition of pain in children is even more problematic due to large differences in their developmental stages; for example, young children have difficulty verbalizing pain.^8^ Older children may hide their pain or be reluctant to ask nurses for help in painful situations.^13^ Therefore, children’s pain should be viewed as a multifaceted concept in which anxiety and fear are central.^8,11^

Various validated self-assessment scales can be used to measure preschool and school-age children’s fear (eg, Children’s Fear Scale [CFS]),^14^ pain (eg, Faces Pain Scale [FPS]),^15^ and anxiety (eg, modified Yale Preoperative Anxiety Scale [mYPAS]), which were developed to help an outside observer assess a child’s anxiety before and after a procedure. The mYPAS measures a child’s activity, voice, emotional expressiveness, and parental need.^16^ Children’s anxiety, fear, and pain can also be objectively assessed using physiological indicators (eg, changes in heart rate, blood pressure, respiratory rate, and oxygen saturation^17^).

Every child has an ethical right to pain relief and its related benefits in health care; nevertheless, studies suggest that children receive limited pain treatment.^17^ Distraction-based interventions represent an essential non-pharmacological method for relieving preschool and school-age children’s anxiety, fear, and pain in the hospital,^18^ for which there is also available research on effectiveness.^1,2,4,5,18–21^ Distraction-based interventions focus the child’s attention on some meaningful activity and can be categorized as active or passive. Active distraction-based interventions encourage children to participate in activities and display their skills during painful procedures, activating their visual, auditory, and kinesthetic senses^1,2,4,5^ (eg, use of virtual reality, interactive toys,^22^ or humor with a hospital clown^19,20^). In contrast, in passive distraction-based interventions, children are not encouraged to participate in activities; instead, they passively follow various interventions that activate their senses^1,2,4,5^ (eg, listening to music, or watching television or videos without the parent or nurse activating the child).^22^ According to some studies of active distraction-based interventions,^1,2,5^ this method more effectively relieved hospitalized children’s anxiety, fear, and pain during venous blood sampling than passive distraction-based intervention.

No systematic reviews have been conducted to evaluate the effectiveness of active and passive distraction-based interventions in relieving hospitalized preschool and school-age children’s anxiety, fear, and pain during venous blood sampling. At present, 6 systematic reviews have focused on the effectiveness of non-pharmacological methods in relieving treatment-related pain, anxiety, and/or distress among hospitalized children undergoing needle-related procedures.^3,18,20,21,23,24^ Systematic reviews have covered children and adolescents from birth to 21 years of age. The findings suggest that non-pharmacological interventions, such as distraction,^18,21,23,24^ hypnosis,^19^ clowns,^20^ vibratory stimulation,^3^ positioning, sucrose, and cold application,^24^ may help relieve pediatric pain, anxiety, and/or distress based on child, parent, and observer reports. It has been suggested that these techniques could be more effective if they considered the child’s age as well as their mental and physical conditions.^21^ Digital distraction can provide modest pain and distress reduction for children undergoing painful procedures (eg, venipuncture, dental, and burn treatments); however, there is no empirical evidence that clearly demonstrates that digital distraction methods are superior to other distraction methods.^18^

A preliminary search was conducted in CINAHL, the Cochrane Database of Systematic Reviews, PROSPERO, and *JBI Evidence Synthesis*, but no current or in-progress systematic reviews on the topic were identified. Five systematic reviews^3,20,21,23,24^ were conducted between 2013 and 2022. One systematic review evaluated the effectiveness of digital technology in distracting children to relieve acute pain.^18^

It is necessary to synthesize how effective active compared with passive distraction-based interventions are at relieving preschool and school-age children’s anxiety, fear, and pain during venous blood sampling to inform the design of future distraction-based interventions.

## Review question

What is the effectiveness of active versus passive distraction-based interventions in relieving hospitalized preschool and school-age children’s anxiety, fear, and pain during venous blood sampling?

## Inclusion criteria

### Participants

This review will include studies that include preschool and school-age children ranging from 2 to 12 years old who were subjected to venous blood sampling during their hospitalization. The cause or underlying disease of hospitalized children’s treatment may also vary in the included studies, and all underlying diseases and causes will be eligible for inclusion. The international definition of preschool age is 2 to 5 years,^25^ and that of school-age children is 6 to 12 years.^26^

### Interventions

The review will include, but not be limited to, studies that evaluate active distraction-based interventions (eg, use of virtual reality, interactive toys,^22^ humor with a hospital clown^19,20^) provided during venous blood sampling.

### Comparator

The review will include studies that compare active distraction-based interventions with passive distraction-based interventions (eg, listening to music, or watching television or videos without the parent or nurse activating the child)^22^ during venous blood sampling. In passive distraction-based interventions, children are not encouraged to participate in activities; instead, they passively follow various interventions that activate their senses.^1,2,4,5^

### Outcomes

This review will include studies with any of the following outcomes: child’s anxiety, fear, and pain, measured using validated anxiety, fear, and pain assessment scales (eg, mYPAS,^16^ CFS,^14^ FPS^15^) and/or behavioral indicators (eg, cry duration time, facial expressions). Secondary outcomes will include physiological indicators (changes in heart rate, respiratory rate per minute, blood pressure, and oxygen saturation). Articles that include only secondary outcomes will also be included in the review.

### Types of studies

This review will include randomized controlled trials and quasi-experimental studies.

## Methods

This systematic review will be conducted in accordance with the JBI methodology for systematic reviews of effectiveness.^25^ This protocol is registered in PROSPERO (CRD42023455617).

### Search strategy

The search strategy will identify published and unpublished studies. Based on the JBI methodology for systematic reviews, a 3-step search strategy will be used for this review.^27^ An initial, limited search of CINAHL (EBSCOhost) and PubMed was conducted to identify articles on the topic, followed by an analysis of the words in the identified articles’ titles and abstracts and the search terms used to describe the article. A second search including all of the identified keywords and search terms will be conducted across all databases. The search strategy developed for CINAHL (EBSCOhost; see Appendix I) will be adapted for each included database and/or information source. Third, the reference lists of all included sources of evidence will be screened for additional relevant studies. Only studies published in English, Swedish, and Finnish will be included due to resource limitations. There will be no restrictions regarding the date of publication.

The databases to be searched will include CINAHL (EBSCOhost), MEDLINE (PubMed), Scopus, and the Cochrane Central Register of Controlled Trials. In-progress and unpublished studies will be searched for in the first 20 records in MedNar, Google Scholar, and PsycEXTRA (APA).

### Study selection

Following the search, all identified citations will be collated and uploaded into Covidence (Veritas Health Innovation, Melbourne, Australia), after which duplicates will be removed. Following a pilot test with 20 citations, 2 independent reviewers will screen titles and abstracts for assessment against the inclusion criteria for the review. The full-text versions of potentially relevant studies will be retrieved, and their citation details imported into Covidence. Two or more independent reviewers will then assess these full-text articles in detail against the inclusion criteria. Any disagreements that arise between the reviewers at each stage of the selection process will be resolved through discussion or with an additional reviewer. The results of the search and the study inclusion process will be reported in full in the final systematic review and presented in a Preferred Reporting Items for Systematic Reviews and Meta-Analyses (PRISMA) flow diagram.^28^

### Assessment of methodological quality

Two independent reviewers will critically appraise all of the eligible studies for methodological quality using the JBI critical appraisal instruments for randomized controlled trials and quasi-experimental studies.^27,29^ Authors will be contacted for missing information or additional data, where required. Any disagreements that arise will be resolved through discussion or with a third reviewer. The results of the critical appraisal will be reported in narrative format and in a table. All studies, regardless of evaluations of their methodological quality, will undergo data extraction and synthesis, where possible. The quality of the studies will be considered in the interpretation of results.

### Data extraction

Two independent reviewers will extract data from the studies included in the review using the standardized data collection tool in Covidence. The extracted data will include detailed information about the participants (eg, the child’s age; gender; previous experiences with venous blood sampling; health status; and physical, cognitive, emotional, and social development), research methods, interventions used during venous blood sampling (eg, active distraction-based intervention, type of comparison), and outcomes (eg, scores on anxiety, fear, and pain scales). Behavioral (eg, duration of crying in seconds; changes in facial expressions, such as frowning) and physiological indicators (changes in heart rate in minutes, changes in upper and lower blood pressure, changes in respiratory rate in minutes, and oxygen saturation in per cent) will be used to assess differences between the intervention and control groups. Attempts will be made to contact the research team twice if any data are missing from a study.

### Data synthesis

Studies will, whenever possible, be pooled in a statistical meta-analysis. Effect sizes will be expressed as weighted (or standardized) final post-intervention mean differences (for continuous data). The corresponding 95% CI will also be calculated and presented. Heterogeneity will be statistically assessed using the standard χ^2^ and *I* ^2^ tests. If data permit, reasons for heterogeneity will be explored using subgroup analysis to examine the level of consistency or significant variations in the treatment effect size across various patient categories (eg, age or gender groups). Where data permit, sensitivity analyses will also be conducted by excluding studies of poor methodological quality to assess the robustness of the conclusions. Statistical analyses will be conducted using fixed-effects or random-effects models for meta-analysis based on guidance from Tufanaru *et al.* ^30^ A funnel plot (Egger test, Begg test, Harbord test) will be generated to assess publication bias if 10 or more studies are included in a meta-analysis. If statistical analysis is not possible, the findings will be reported narratively.

### Assessing certainty in the findings

The Grading of Recommendations Assessment, Development, and Evaluation (GRADE) approach will be used to assess certainty in the quality of evidence.^31^ The results of this assessment will be shown in a Summary of Findings created using GRADEpro software (McMaster University, ON, Canada). The Summary of Findings will include the following outcomes identified during venous blood sampling:

- anxiety, assessed using various measures (eg, the mYPAS)^16^
- fear, assessed using a range of measures (eg, the CFS)^14^
- pain, measured using appropriate tools (eg, the FPS).^15^

Children’s anxiety, fear, and pain during venous blood sampling can also be assessed using behavioral indicators (eg, cry duration time, facial expressions) and changes in physiological indicators (heart rate, respiratory rate per minute, blood pressure, and oxygen saturation). The Summary of Findings will present the following information where appropriate: absolute risks in treatment and control; estimates of relative risk; and a ranking of the quality of evidence based on study limitations, indirectness, inconsistency, imprecision, and publication bias.

## Acknowledgments

Sirpa Grekula at the Oulu University Medical Library for support with the database searches.

## Author contributions

SH, AKP, and TP were responsible for the systematic review protocol conception and design. SH performed the data collection from the CINAHL (EBSCOhost) database. SH, AKP, TP, and AMT were responsible for drafting the manuscript. AKP, TP, and AMT made critical revisions to the manuscript for content. TP supervised the protocol. All authors have accepted the final version.

## Appendix I: Search strategy

### CINAHL (EBSCOhost)

Search conducted: September 28, 2023

**Table:**

Search	Query	Records retrieved
S1	( ( ( (MH “Child+”) OR (MH “Pediatric Nursing+”) OR (MH “Pediatric Care+”) ) OR ( child* OR pediatric* OR paediatric* ) ) ) AND ( ( (MH “Blood Specimen Collection+”) OR ( “blood sampl*” OR “blood draw*” OR “blood specimen collect*” OR “arterial puncture*” OR venipunctur* OR phlebotomy OR intravenous OR canullation* OR “blood taking” ) ) ) AND ( ((MH “Multimedia”) OR ( (MH “Augmented Reality”) OR ( (MH “Play and Playthings+”) OR (MH “Virtual Reality+”) OR (MH “Videorecording+”) OR (MH “Music”) OR (MH “Television”) OR (MH “Vibration”) OR (MH “Sound+”) OR (MH “Voice+”)) ) OR ( “virtual reality” OR “augmented reality” OR “extended reality” OR “mixed reality” OR toy* OR music OR television OR video* OR play* OR game* OR gaming OR multimedia OR clown* OR “VR glasses” OR vibrat* OR sound* OR voice* ) OR ( ( (MH “Distraction”) OR distract*) )) ) AND ( ( ( (MH “Fear”) OR (MH “Anxiety+”) OR (MH “Pain+”) OR ( fear* OR pain* OR anxiety OR scared OR distress*) ) )	421

Language limits: English, Finnish, Swedish

All search terms were searched as subject headings and free words.
